# Progress Toward Regional Measles Elimination — Worldwide, 2000–2020

**DOI:** 10.15585/mmwr.mm7045a1

**Published:** 2021-11-12

**Authors:** Meredith G. Dixon, Matt Ferrari, Sebastien Antoni, Xi Li, Allison Portnoy, Brian Lambert, Sarah Hauryski, Cynthia Hatcher, Yoann Nedelec, Minal Patel, James P. Alexander, Claudia Steulet, Marta Gacic-Dobo, Paul A. Rota, Mick N. Mulders, Anindya S. Bose, Alexander Rosewell, Katrina Kretsinger, Natasha S. Crowcroft

**Affiliations:** ^1^Global Immunization Division, Center for Global Health, CDC; ^2^Center for Infectious Disease Dynamics, The Pennsylvania State University, University Park, Pennsylvania; ^3^Department of Immunization, Vaccines, and Biologicals, World Health Organization, Geneva, Switzerland; ^4^Center for Health Decision Science, Harvard T.H. Chan School of Public Health, Boston, Massachusetts; ^5^Division of Viral Diseases, National Center for Immunization and Respiratory Diseases, CDC.

In 2012, the World Health Assembly endorsed the Global Vaccine Action Plan,* with the objective of eliminating measles^†^ in five of the six World Health Organization (WHO) regions by 2020 (*1*). The Immunization Agenda 2021–2030 (IA2030)^§^ uses measles incidence as an indicator of the strength of immunization systems. The Measles-Rubella Strategic Framework 2021–2030^¶^ and the Measles Outbreaks Strategic Response Plan 2021–2023** are aligned with the IA2030 and highlight robust measles surveillance systems to document immunity gaps, identify root causes of undervaccination, and develop locally tailored solutions to ensure administration of 2 doses of measles-containing vaccine (MCV) to all children. This report describes progress toward World Health Assembly milestones and measles elimination objectives during 2000–2020 and updates a previous report (*2*). During 2000–2010, estimated MCV first dose (MCV1) coverage increased globally from 72% to 84%, peaked at 86% in 2019, but declined to 84% in 2020 during the COVID-19 pandemic. All countries conducted measles surveillance, although fewer than one third achieved the sensitivity indicator target of ≥2 discarded^††^ cases per 100,000 population in 2020. Annual reported measles incidence decreased 88% during 2000–2016, from 145 to 18 cases per 1 million population, rebounded to 120 in 2019, before falling to 22 in 2020. During 2000–2020, the annual number of estimated measles deaths decreased 94%, from 1,072,800 to 60,700, averting an estimated 31.7 million measles deaths. To achieve regional measles elimination targets, enhanced efforts are needed to reach all children with 2 MCV doses, implement robust surveillance, and identify and close immunity gaps.

## Immunization Activities

WHO and UNICEF estimate immunization coverage using data from administrative records (calculated by dividing the number of vaccine doses administered by the estimated target population, reported annually), country estimates, and vaccination coverage surveys to estimate MCV1 and second dose MCV (MCV2) coverage through routine immunization (i.e., not mass campaigns).^§§^ During 2000–2010, estimated MCV1 coverage worldwide increased from 72% to 84%. However, coverage stagnated at 84%–85% since 2010, peaked at 86% in 2019, and declined to 84% in 2020. Regional variation exists; however, five of the six WHO regions reported a decline in MCV1 coverage between 2019 and 2020 (Table 1).

**TABLE 1:** Estimates of coverage with the first and second doses of measles-containing vaccine administered through routine immunization services, reported measles cases, and incidence, by World Health Organization region — worldwide, 2000, 2010, 2016, 2019, and 2020

WHO region/Year (no. of countries in region)	%				No. of reported measles cases^§^ (% of total cases)	Measles incidence per 1 million population^§,¶^
	MCV1* coverage	Countries with ≥90% MCV1 coverage^†^	MCV2* coverage	Reporting countries with <5 measles cases per 1 million population^§^		
**African**						
2000 (46)	53	9	5	8	520,102 (60.9)	842
2010 (46)	73	37	4	30	199,174 (57.9)	235
2016 (47)	69	34	22	51	36,269 (27.4)	37
2019 (47)	70	30	33	34	618,595 (70.9)	567
2020 (47)	68	15	36	32	115,364 (77.0)	108
**Americas**						
2000 (35)	93	63	65	89	1,754 (0.2)	2
2010 (35)	93	74	67	100	247 (0.1)	0.3
2016 (35)	92	66	80	100	97 (0.1)	0.1
2019 (35)	87	69	72	91	21,971 (2.5)	32
2020 (35)	85	37	73	100	1,548 (1.0)	2
**Eastern Mediterranean**						
2000 (21)	71	57	28	17	38,592 (4.5)	90
2010 (21)	77	62	52	40	10,072 (2.9)	17
2016 (21)	82	57	74	55	6,275 (4.7)	10
2019 (21)	84	52	75	42	18,458 (2.1)	27
2020 (21)	83	33	76	64	6,122 (4.1)	10
**European**						
2000 (52)	91	62	48	45	37,421 (4.4)	50
2010 (53)	93	83	80	69	30,625 (8.9)	34
2016 (53)	93	81	88	82	4,440 (3.4)	5
2019 (53)	96	85	91	29	106,130 (12.2)	116
2020 (53)	94	57	91	80	10,772 (7.2)	17
**South-East Asia**						
2000 (10)	63	30	3	0	78,558 (9.2)	51
2010 (11)	83	45	15	36	54,228 (15.8)	30
2016 (11)	89	64	75	27	27,530 (20.8)	14
2019 (11)	94	73	83	30	29,389 (3.4)	15
2020 (11)	88	55	78	56	9,389 (6.3)	5
**Western Pacific**						
2000 (27)	85	48	2	30	177,052 (20.7)	104
2010 (27)	96	63	87	68	49,460 (14.4)	27
2016 (27)	96	63	93	68	57,879 (43.7)	31
2019 (27)	95	67	93	46	78,479 (9.0)	41
2020 (27)	95	44	94	60	6,601 (4.4)	4
**Total**						
**2000 (191)**	**72**	**45**	**18**	**38**	**853,479 (100)**	**145**
**2010 (193)**	**84**	**63**	**42**	**60**	**343,806 (100)**	**50**
**2016 (194)**	**85**	**61**	**67**	**70**	**132,490 (100)**	**18**
**2019 (194)**	**86**	**62**	**71**	**45**	**873,022 (100)**	**120**
**2020 (194)**	**84**	**39**	**70**	**65**	**149,796 (100)**	**22**

**Abbreviations:** MCV1 = first dose of measles-containing vaccine; MCV2 = second dose of measles-containing vaccine; WHO = World Health Organization.

* https://www.who.int/teams/immunization-vaccines-and-biologicals/immunization-analysis-and-insights/global-monitoring/immunization-coverage/who-unicef-estimates-of-national-immunization-coverage; data accessed July 6, 2021.

^†^ Denominator is the number of WHO member states.

^§^ https://immunizationdata.who.int/pages/incidence/measles.html?GROUP%20=%20Countries&YEAR%20=; data accessed July 6, 2021. Only those countries that reported data are in the numerator and denominator.

^¶^ Population data from United Nations, Department of Economic and Social Affairs, Population Division, 2020. Any country not reporting measles cases for that year was removed from both the numerator and denominator in calculating incidence.

Among 194 WHO member states, 75 (39%) achieved ≥90% MCV1 coverage in 2020, a 13% decrease from 86 (45%) countries in 2000, and a 37% decrease from 119 (61%) countries in 2019. In 2020, 22.3 million infants did not receive MCV1 through routine immunization services, an increase of three million (16%) from 2019. The 10 countries with the highest numbers of infants not receiving MCV1 were Nigeria (3.3 million), India (2.6 million), the Democratic Republic of the Congo (1.5 million), Ethiopia (1.4 million), Indonesia (1.1 million), Pakistan (1.0 million), Angola (0.7 million), the Philippines (0.6 million), Brazil (0.6 million), and Afghanistan (0.4 million); accounting for nearly two thirds (59%) of the global total. Estimated global MCV2 coverage nearly quadrupled from 18% in 2000 to 71% in 2019, then declined to 70% in 2020. The number of countries offering MCV2 increased 88%, from 95 (50%) in 2000 to 179 (92%) in 2020. Two countries (Madagascar and Nigeria) introduced MCV2 in 2020.

Approximately 36 million persons received MCV during supplementary immunization activities (SIAs)^¶¶^ in 24 countries in 2020. An additional two million persons received MCV during measles outbreak response activities. Twenty-four SIAs in 23 countries planned for 2020 were postponed because of the COVID-19 pandemic, affecting ≥93 million persons (LL Ho, WHO, personal communication, November 2021).

## Reported Measles Incidence and Surveillance Performance

In 2020, all 194 countries conducted measles surveillance, and 193*** (99%) had access to standardized quality-controlled laboratory testing through the WHO Global Measles and Rubella Laboratory Network (GMRLN).^†††^ In spite of this access, surveillance worsened in 2020: GMRLN received 122,517 specimens for measles testing in 2020, the lowest number since 2010, and only 46 (32%) of 144 countries that reported discarded cases achieved the sensitivity indicator target of two or more discarded cases per 100,000 population, compared with 81 (52%) of 157 countries in 2019.

Countries report the number of incident measles cases^§§§^ to WHO and UNICEF annually, using the Joint Reporting Form.^¶¶¶^ During 2000–2016, the number of reported measles cases decreased 84%, from 853,479 (2000) to 132,490 (2016). From 2000 to 2016, annual measles incidence decreased 88%, from 145 cases per million (2000) to 18 (2016), then increased 567% to 120 per million (2019) before decreasing 82% to 22 (2020) (Table 1). In 2020, 26 large and disruptive outbreaks (≥20 cases per million) were reported across five WHO regions (Supplementary Table, https://stacks.cdc.gov/view/cdc/111172); 17 (65%) of these outbreaks occurred in countries in the African Region (AFR).

Genotypes of viruses isolated from persons with measles were reported by 47 (46%) of 102 countries reporting at least one measles case in 2020, compared with 88 (62%) of 141 countries in 2019. The number of genotypes detected per year decreased from 13 in 2002, to three in 2020, a sign of progress toward elimination. Among 1,268 reported sequences in 2020, 947 (75%) were D8, 307 (24%) were B3, and 14 (1%) were D4.

## Measles Case and Mortality Estimates

A previously described model for estimating measles cases and deaths (*3*) was updated with annual vaccination coverage data, case data, and United Nations population estimates for all countries during 2000–2020. The model was revised (*4*,*5*) to incorporate alternative assumptions of correlation between routine MCV doses and SIAs and updated case-fatality ratios, enabling derivation of new global disease and mortality estimates. On the basis of updated annual data and model revisions, the estimated number of measles cases decreased 79%, from 36,763,000 in 2000 to 7,549,000 in 2020; estimated annual measles deaths decreased 94%, from 1,072,800 to 60,700 (Table 2). During 2000–2020, compared with no measles vaccination, measles vaccination prevented an estimated 31.7 million deaths globally (Figure).

**TABLE 2:** Estimated number of measles cases and deaths,* by World Health Organization region — worldwide, 2000 and 2020

WHO region/Year (no. of countries in region)	Estimated no. of measles cases (95% CI)	Estimated no. of measles deaths (95% CI)	Estimated % measles mortality reduction, 2000–2020	Cumulative no. of measles deaths averted by vaccination, 2000–2020
**African**				
2000 (46)	11,416,700 (7,212,400–16,519,900)	647,800 (429,500–919,300)	95	16,129,100
2020 (47)	1,944,700 (1,227,300–3,482,200)	33,400 (22,300–56,000)		
**Americas**				
2000 (35)	8,800 (4,400–35,000)	NA^†^	NA	105,200
2020 (35)	43,700 (21,800–174,700)	NA^†^		
**Eastern Mediterranean**				
2000 (21)	4,641,600 (2,120,400–10,419,900)	156,400 (83,400–317,500)	87	3,274,300
2020 (21)	2,043,600 (1,394,300–2,944,600)	20,400 (14,400–28,700)		
**European**				
2000 (52)	813,500 (592,400–1,296,000)	4,100 (3,000–5,400)	97	103,400
2020 (53)	179,600 (70,800–392,500)	100 (0–200)		
**South-East Asia**				
2000 (10)	13,856,500 (10,730,400–17,563,500)	231,400 (190,500–282,000)	98	10,487,700
2020 (11)	2,552,600 (1,509,300–3,902,300)	5,600 (3,800–8,000)		
**Western Pacific**				
2000 (27)	6,026,000 (4,955,600–7,899,400)	33,100 (26,700–38,200)	96	1,597,700
2020 (27)	784,900 (153,700–2,173,500)	1,200 (300–2,800)		
**Total**				
**2000 (191)**	**36,763,000 (25,615,600–53,733,800)**	**1,072,800 (733,100–1,562,300)**	**94**	**31,697,500**
**2020 (194)**	**7,549,000 (4,377,300–13,069,700)**	**60,700 (40,800–95,800)**		

**Abbreviations:** NA = not applicable; WHO = World Health Organization.

* The measles mortality model used to generate estimated measles cases and deaths is rerun each year using the new and revised annual WHO/UNICEF estimates of national immunization coverage data, as well as updated surveillance data. In addition, in 2021, the model was revised with respect to correlations in coverage among different measles-containing vaccine delivery methods; therefore, the estimated number of cases and mortality estimates in this report differ from previous reports.

^†^ Estimated measles mortality was too low to allow reliable measurement of mortality reduction.

**FIGURE:**
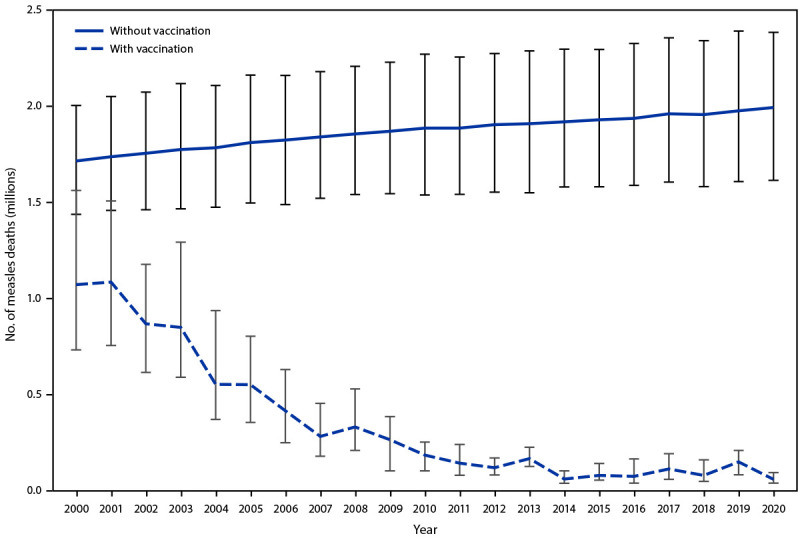
Estimated number of annual measles deaths with vaccination and without vaccination* — worldwide, 2000–2020 * Deaths prevented by vaccination are estimated by the area between estimated deaths with vaccination and without vaccination (total of 31.7 million deaths prevented during 2000–2020). Vertical bars represent upper and lower 95% CIs around the point estimate.

## Regional Verification of Measles Elimination

By the end of 2020, 81 (42%) countries had been verified by independent regional commissions as having sustained measles elimination, but no new countries had achieved elimination. No WHO region had achieved and sustained elimination, and no AFR country has yet been verified to have eliminated measles. The WHO Region of the Americas achieved verification of measles elimination in 2016; however, endemic measles transmission was reestablished in Venezuela (2016) and Brazil (2018). Since 2016, endemic transmission has been reestablished in nine other countries that had previously eliminated measles (Albania, Cambodia, Czechia, Germany, Lithuania, Mongolia, Slovakia, the United Kingdom, and Uzbekistan).

## Discussion

A substantial decrease in measles incidence and associated mortality occurred worldwide during 2000–2016, followed by a global resurgence during 2017–2019, then an apparent decline in 2020 during the COVID-19 pandemic. Despite this decline, millions more children were susceptible to measles at the end of 2020 than in 2019. MCV1 coverage decreased globally and in all but one region in 2020; 22.3 million children did not receive MCV1 through routine immunization, and at least 93 million persons did not receive MCV because of COVID-19–related postponement of measles SIAs. Measles surveillance also deteriorated in 2020: the number of specimens submitted was the lowest in over a decade, many countries did not report, and few countries (32%) achieved the measles surveillance sensitivity indicator. Increased population susceptibility and suboptimal measles surveillance portend an immediate elevated risk for measles transmission and outbreaks, threatening the already fragile progress toward regional elimination goals.

The extent to which measles transmission declined in 2020 is unclear. Fewer reported cases might reflect lower transmission secondary to increased immunity from outbreaks during 2017–2019, COVID-19 mitigation measures, or both. Conversely, measles cases might have been underreported in 2020 because of reductions in health care–seeking behavior from patients, health facility availability and reporting, or overall pandemic-related health system disruptions. Large and disruptive measles outbreaks in 2020, however, suggest that measles transmission was underreported. Robust case-based measles surveillance systems enable countries to detect and respond promptly to measles cases and outbreaks. Expanded virologic surveillance can better monitor local patterns of transmission, particularly in high-incidence areas like AFR. The Measles Outbreaks Strategic Response Plan 2021–2023 recommends annual risk assessments to strengthen preparedness and response, investigation of every outbreak, rapid implementation of effective interventions to stop transmission, and root cause analysis to close immunity gaps and prevent future outbreaks through tailored approaches.

Coverage of ≥95% with MCV1 and MCV2 is necessary to ensure and sustain high population immunity against measles. MCV1 coverage has stagnated since 2010, and the largest annual increase since 2000 in children who did not receive MCV1 was reported in 2020, representing an acute setback in progress toward measles elimination (*6*). Accelerated efforts are needed to expand MCV1 coverage among the 22.3 million unvaccinated children in 2020 and ensure immunization of future birth cohorts. Routine MCV2 immunization has been recommended since 2017 (*7*); timely introduction is needed in the 15 countries that have yet to introduce MCV2, including 13 of the 47 countries in AFR. The revised measles estimation model indicates that in many countries, MCV is provided through SIAs to children with access to routine services (*4*); instead, SIAs should aim to fill immunity gaps among persons without access to routine service delivery, including older children and adults.

The findings in this report are subject to at least three limitations. First, in 2020, 35 (18%) countries did not report MCV1 coverage and 50 (26%) did not report case data to WHO/UNICEF by the deadline. This decreased reporting precludes a complete understanding of measles epidemiology globally and regionally. Second, revisions to the measles estimation model limit comparability of the estimates in this report to those of previous years’ reports. Finally, genotype data are based on a limited number of sequences, most of which do not originate from AFR, which has the highest disease incidence. The proportion of circulating genotypes might differ from those reported here.

Progress toward measles elimination during the COVID-19 pandemic and beyond necessitates strong case-based surveillance systems to document immunity gaps and quickly identify cases and outbreaks. Outbreaks should be viewed as opportunities to identify weaknesses across the immunization system and develop tailored strategies to close immunity gaps. Together, these actions will bolster measles elimination efforts while strengthening immunization systems.

**Summary**

**What is already known about this topic?**

All six World Health Organization (WHO) regions remain committed to measles elimination.

**What is added by this report?**

Annual reported measles incidence decreased globally during 2000–2016, increased in all regions during 2017–2019, then decreased in 2020. Measles surveillance, already suboptimal, worsened in 2020. Since 2000, estimated measles deaths decreased 94%. Measles vaccination has prevented an estimated 31.7 million deaths worldwide. No WHO region has achieved and maintained measles elimination.

**What are the implications for public health practice?**

To achieve regional measles elimination targets, enhanced efforts are needed to reach all children with 2 doses of measles-containing vaccine, implement robust surveillance, and identify and close immunity gaps.

## Footnotes

* The Global Vaccine Action Plan is the implementation plan of the Decade of Vaccines, a collaboration between WHO; UNICEF; the Bill and Melinda Gates Foundation; the National Institute of Allergy and Infectious Diseases; the African Leaders Malaria Alliance; Gavi, the Vaccine Alliance; and others to extend the full benefit of immunization to all persons by 2020 and beyond. In addition to 2015 targets, it also set a target for measles and rubella elimination in five of the six WHO regions by 2020. https://www.who.int/teams/immunization-vaccines-and-biologicals/strategies/global-vaccine-action-plan

† Measles elimination is defined as the absence of endemic measles virus transmission in a region or other defined geographic area for ≥12 months in the presence of a high-quality surveillance system that meets targets of key performance indicators.

§ Immunization Agenda 2030 is the global vision and strategy to extend the benefits of vaccines to everyone, everywhere, developed by immunization stakeholders and endorsed by the World Health Assembly in 2020. https://www.who.int/teams/immunization-vaccines-and-biologicals/strategies/ia2030

¶ The Measles and Rubella Strategic Framework 2021–2030 aims to provide high-level guidance for developing regional and national strategies and operational plans. It was developed through a broad consultative process that generated feedback on achievements and major shortfalls in measles and rubella control over the past decade and defined strategic pivots and focus areas for the next decade. It is meant to serve as a disease-specific strategy under the IA2030 structure, and it aligns with other important strategy documents, including WHO’s Thirteenth General Programme of Work 2019–2023; the UNICEF Immunization Roadmap 2018–2030; and Gavi, the Vaccine Alliance’s 2021–2025 strategy. The Measles and Rubella Strategic Framework 2021–2030 envisions “a world free from measles and rubella.” https://www.who.int/publications/i/item/measles-and-rubella-strategic-framework-2021-2030

** The Measles Outbreaks Strategic Response Plan 2021–2023 envisions a world in which all countries are equipped with robust measles outbreak prevention, preparedness, and response systems and contains four objectives: 1) improved coordination mechanisms for measles outbreak preparedness and response; 2) expanded vaccination efforts in vulnerable communities through resource mobilization for risk-based national plans in countries that are not eligible for Gavi support; 3) enhanced national capacity for outbreak preparedness in priority countries (e.g., planning, detection, readiness for investigation, and response), including robust surveillance; and 4) improved timeliness and effectiveness of investigation and response to measles outbreaks, including detection, root cause analysis to identify programmatic gaps to prevent future outbreaks, after action reviews and recovery. https://apps.who.int/iris/handle/10665/340657

†† A discarded case is defined as a suspected case that has been investigated and determined to be neither measles nor rubella using 1) laboratory testing in a proficient laboratory or 2) epidemiologic linkage to a laboratory-confirmed outbreak of a communicable disease that is not measles or rubella. The discarded case rate is used to measure the sensitivity of measles surveillance.

§§ Calculated for MCV1, among children aged 1 year or, if MCV1 is given at age ≥1 year, among children aged 24 months. Calculated for MCV2 among children at the recommended age for administration of MCV2, per the national immunization schedule. WHO/UNICEF estimates of national immunization coverage are available at https://www.who.int/teams/immunization-vaccines-and-biologicals/immunization-analysis-and-insights/global-monitoring/immunization-coverage/who-unicef-estimates-of-national-immunization-coverage.

¶¶ SIAs generally are carried out using two target age ranges. An initial, nationwide catch-up SIA focuses on all children aged 9 months–14 years, with the goal of eliminating susceptibility to measles in the general population. Periodic follow-up SIAs then focus on all children born since the last SIA. Follow-up SIAs generally are conducted nationwide every 2–4 years and focus on children aged 9–59 months; their goal is to eliminate any measles susceptibility that has developed in recent birth cohorts because of low MCV coverage and to protect children who did not respond to MCV1. Data on SIAs by country are available at https://immunizationdata.who.int/listing.html?topic=&location=.

*** São Tomé and Príncipe did not have access to standardized quality-controlled testing by the WHO Measles and Rubella Laboratory Network in 2020.

††† http://www.who-measles.org/Public/Web_Front/main.php; data accessed August 3, 2021.

§§§ https://apps.who.int/immunization_monitoring/globalsummary/timeseries/tsincidencemeasles.html; accessed July 6, 2020. Only countries that reported data are in both the numerator and denominator.

¶¶¶ https://immunizationdata.who.int/pages/incidence/measles.html?GROUP%20=%20Countries&YEAR%20=. Countries that did not report case data, by year (total number, country name), are: 2000 (25: Algeria, Austria, Belgium, Comoros, Equatorial Guinea, Fiji, Finland, Germany, Guinea-Bissau, Ireland, Libya, Mauritania, Monaco, Montenegro, North Korea, Samoa, Saudi Arabia, Seychelles, Slovenia, Solomon Islands, South Sudan, Switzerland, Timor-Leste, Tuvalu, and Yemen); 2010 (five: Federated States of Micronesia, Libya, Monaco, Nauru, and South Sudan); 2016 (15: Belgium, Cabo Verde, Cook Islands, Haiti, Italy, Kiribati, Marshall Islands, Monaco, Morocco, Mozambique, Niue, Samoa, Singapore, Tuvalu, and Vanuatu); 2019 (nine: Austria, Djibouti, Marshall Islands, Morocco, North Korea, Palau, Solomon Islands, Switzerland, and the United States); 2020 (50: Afghanistan, Albania, Algeria, Bahamas, Belgium, Bhutan, Bosnia and Herzegovina, Brazil, Cabo Verde, Cook Islands, Cyprus, Czechia, Djibouti, Federated States of Micronesia, Fiji, Gambia, Germany, Iraq, Kuwait, Kyrgyzstan, Libya, Marshall Islands, Mauritius, Monaco, Montenegro, Morocco, Namibia, Nauru, Netherlands, Niue, North Macedonia, Oman, Palau, Peru, Poland, Portugal, Korea, Moldova, Saint Kitts and Nevis, São Tomé and Príncipe, Serbia, Singapore, Solomon Islands, Tajikistan, Thailand, Tonga, Trinidad and Tobago, Turkey, Turkmenistan, and Tuvalu). Countries do not provide WHO with their reasons for not reporting case data.
